# Adult neurogenic process in the subventricular zone‐olfactory bulb system is regulated by Tau protein under prolonged stress

**DOI:** 10.1111/cpr.13027

**Published:** 2021-05-14

**Authors:** Chrysoula Dioli, Patrícia Patrício, Lucilia‐Goreti Pinto, Clemence Marie, Mónica Morais, Sheela Vyas, João M. Bessa, Luisa Pinto, Ioannis Sotiropoulos

**Affiliations:** ^1^ School of Medicine Life and Health Sciences Research Institute (ICVS) University of Minho Braga Portugal; ^2^ ICVS/3B's ‐ PT Government Associate Laboratory Braga/Guimarães Portugal; ^3^ Institute of Biology Paris Seine Team Gene Regulation and Adaptive Behaviors Department of Neurosciences Paris Seine Sorbonne Université CNRS UMR 8246 INSERM U1130 Paris France

**Keywords:** chronic stress, neurogenesis, olfactory bulb, oligodendrogenesis, subventricular zone, Tau protein

## Abstract

**Objectives:**

The area of the subventricular zone (SVZ) in the adult brain exhibits the highest number of proliferative cells, which, together with the olfactory bulb (OB), maintains constant brain plasticity through the generation, migration and integration of newly born neurons. Despite Tau and its malfunction is increasingly related to deficits of adult hippocampal neurogenesis and brain plasticity under pathological conditions [e.g. in Alzheimer's disease (AD)], it remains unknown whether Tau plays a role in the neurogenic process of the SVZ and OB system under conditions of chronic stress, a well‐known sculptor of brain and risk factor for AD.

**Materials and methods:**

Different types of newly born cells in SVZ and OB were analysed in animals that lack *Tau* gene (Tau‐KO) and their wild‐type littermates (WT) under control or chronic stress conditions.

**Results:**

We demonstrate that chronic stress reduced the number of proliferating cells and neuroblasts in the SVZ leading to decreased number of newborn neurons in the OB of adult WT, but not Tau‐KO, mice. Interestingly, while stress‐evoked changes were not detected in OB granular cell layer, Tau‐KO exhibited increased number of mature neurons in this layer indicating altered neuronal migration due to Tau loss.

**Conclusions:**

Our findings suggest the critical involvement of Tau in the neurogenesis suppression of SVZ and OB neurogenic niche under stressful conditions highlighting the role of Tau protein as an essential regulator of stress‐driven plasticity deficits.

## Graphical abstract

Despite Tau and its malfunction is increasingly related to deficits of adult brain plasticity under pathological conditions [ e.g. Alzheimer's disease (AD)], it remains unknown whether Tau plays a role in neurogenic process of the subventricular zone‐olfactory bulb system under conditions of chronic stress, a well‐known sculptor of brain and a risk factor for AD. We hereby demonstrate for the first time that loss of Tau blocks the stress‐evoked suppression of proliferating cells and neuroblasts, but not oligodendrocyte progenitors, in the SVZ leading to reduced newborn neurons in olfactory bulb of adult brain.

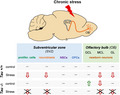

## 1 INTRODUCTION

**FIGURE 1:**
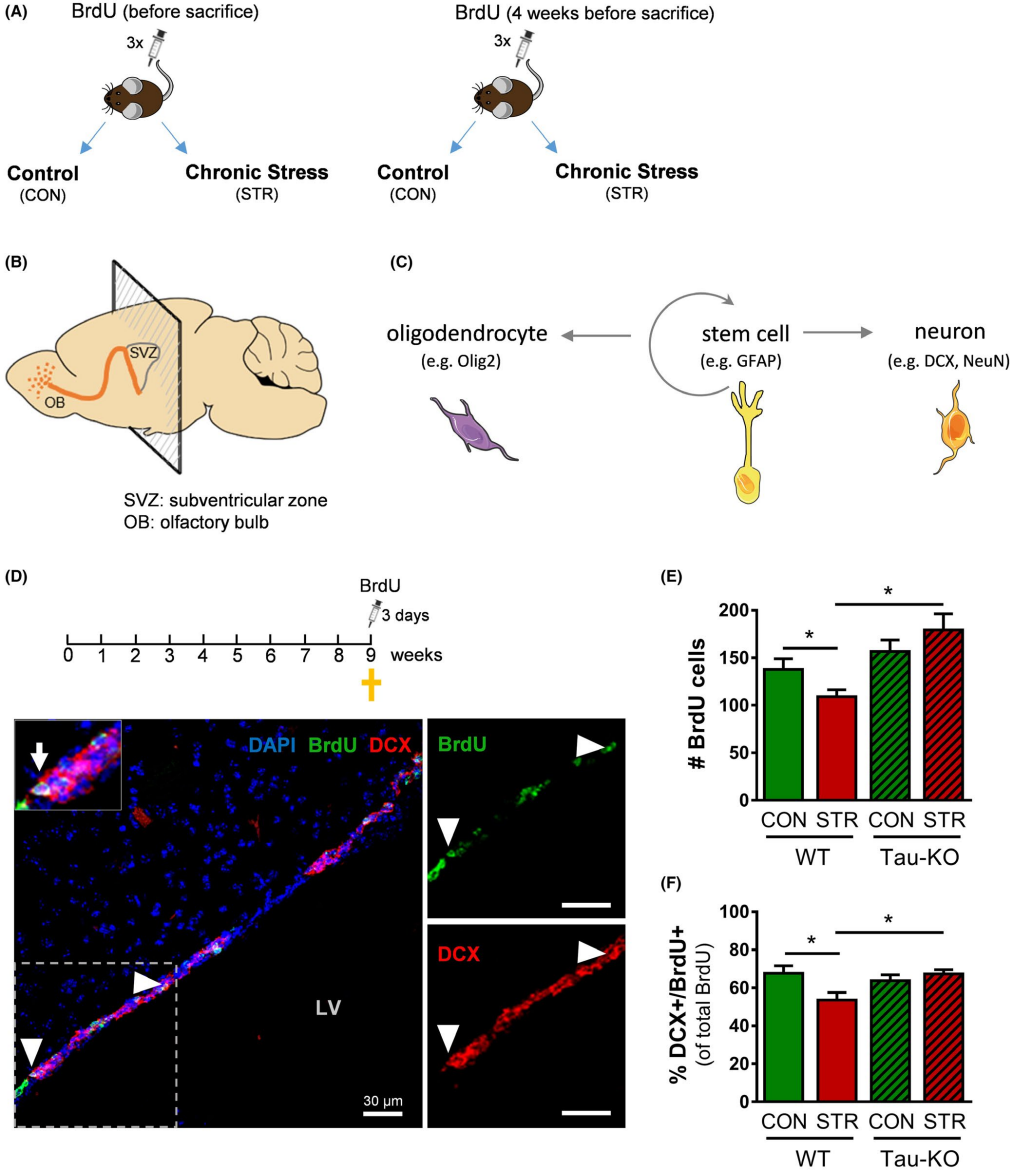
Chronic stress suppresses the number of proliferating cells and neuroblasts in the adult subventricular zone of WT, but not Tau‐KO, animals. A, Schematic representation of the experimental design where wild‐type (WT) and Tau‐knockout (Tau‐KO) mice were divided into control (CON) and chronic stress (STR) groups. Animals of all groups were randomly divided into two groups receiving 5‐bromo‐2′‐deoxyuridine (BrdU) injections before sacrifice (left panel) and 4 weeks before sacrifice (right panel). B,C, Schematic illustration of the mouse brain (B) highlighting the neurogenic areas of the subventricular zone (SVZ) and olfactory bulb (OB) as well as different types of newly born SVZ cells analysed and the markers used for their monitoring. D‐F, Representative microphotograph of BrdU/DCX double‐labelled cells (arrow head) in the SVZ (D). Chronic stress evoked a decrease in BrdU‐positive cell density (reflecting proliferating cells) in WT, but not Tau‐KO, animals. Note that stressed Tau‐KO animals present higher number of proliferating cells when compared to stressed WT animals (E). Similarly, stress reduced the percentage of DCX/BrdU double‐labelled cells (reflecting neuroblasts) only in WT animals (F). All numerical data are shown as mean ± s.e.m (*P < .05). CON, control‐non‐stressed; STR, stressed; BrdU, 5‐bromo‐2′‐deoxyuridine; DCX, doublecortin; WT, wild type; Tau‐KO, Tau‐knockout

The brain is the most adaptive of all organs due to its continuous plasticity in response to a variety of internal and environmental stimuli. A dynamic form of neuronal plasticity in the adult brain is neurogenesis, the process of generation of new, functional neurons from neural stem cells (NSCs) and progenitor cells,^1^ which enables the brain to adapt to the constantly evolving interaction between environmental signals and the brain's internal reaction to these stimuli.^2^ The two main neurogenic niches of the adult brain are the hippocampal dentate gyrus (DG) and the subventricular zone (SVZ) of lateral ventricles^3^; the latter exhibits the biggest amount of proliferative cells in the brain.^4^, ^5^ Newly born neurons generated in the SVZ of the adult brain migrate through the rostral migratory stream to the olfactory bulb (OB) where they differentiate into local circuit interneurons that are implicated in learning and memory processes related to smell sensation in rodents.^6^, ^7^ In brief, type B cells are quiescent neural stem cells (NSCs) expressing glial fibrillary acidic protein (GFAP) that give rise to type C cells (also known as transient‐amplifying progenitors); type C cells give rise to type A cells, which are neuroblasts expressing doublecortin and migrate to the OB.^8^, ^9^ In the OB, these neuroblasts differentiate into interneurons and migrate radially to the outer cell layers, namely granular cell layer (GCL), mitral cell layer (MCL) and glomerular cell layer (GL). Specifically, they differentiate in the GCL and MCL into granule cells (GC) and in the GL into periglomerular cells (PGC).^10^, ^11^ Additionally, it has also been described that type B cells generate oligodendrocytes—see also Figure 1.^12^, ^13^

Although the extent and relevance of adult neurogenesis in humans are currently debated,^14^, ^15^ accumulating evidence suggests that neurogenesis persists in the adult brain of both humans and rodent animals during the entire lifespan while it drops in Alzheimer's disease (AD)^16^, ^17^, ^18^, ^19^, ^20^ and other pathological conditions causally related to AD, such as depression and stress.^21^, ^22^, ^23^ Chronic stress, a major precipitant of depression and AD^24^, ^25^, ^26^, ^27^ is known to impair brain plasticity, including suppression of neurogenesis.^23^, ^28^, ^29^, ^30^, ^31^, ^32^ Recent evidence about stress‐driven neurogenic deficits highlights the critical role for the cytoskeletal Tau protein^22^, ^33^ a prominent stabilizer of microtubules (MT),^34^ which promotes co‐organization of MT and actin networks.^35^, ^36^, ^37^, ^38^ However, our knowledge related to the impact of chronic stress on adult neurogenesis is mainly based on the hippocampus, as the vast majority of studies have neglected the other main neurogenic niche of the adult brain, the SVZ. As a matter of fact, the SVZ area exhibits the highest number of proliferative cells in the adult brain^4^, ^5^ and constitutes the origin of the newly born cells/neurons that migrate into the OB under control conditions^39^ and to other neocortical sites under injury (eg. after stroke or trauma).^40^ Also, hippocampal and SVZ‐OB cytogenic areas exhibit essential differences in their anatomical/layer organization and input received from other brain areas as well as the type of newborn cells generated in each of these two neurogenic niches.^41^ Moreover, their vulnerability to stress or pharmacological/irradiation treatment may be different, as previous studies suggested.^42^, ^43^ Despite that the above findings point towards essential differences between these two neurogenic niches in the adult brain, our knowledge about the cell‐type specific impact on chronic stress on SVZ‐OB system and the underlying mechanisms remain poor.

In light of the limited and conflicting evidence about whether exposure to stressful conditions affects (or not) the SVZ and OB cytogenesis in the adult brain^42^, ^43^ and the selective involvement of Tau in specific types of newborn cells (eg DG newborn neurons, but not glial cells), the current study aims to clarify the effect of stress on different populations of newborn cells in the SVZ‐OB system. For that purpose, we have exposed animals lacking Tau protein (Tau‐KO) and their wild type (WT) littermates to a chronic unpredictable stress (CUS) paradigm and evaluated differences in the cell population resident in the SVZ and OB neurogenic niches. Our findings suggest that exposure to chronic stress suppresses proliferation as well as neuronal differentiation and maturation in the SVZ and OB of the adult brain while the absence of Tau protein diminishes these neuroplastic effects of stress highlighting an essential role for Tau in the mechanisms through which prolonged stressful conditions damage brain plasticity.

## 2 MATERIALS AND METHODS

### 2.1 Animals

Twenty‐eight male mice lacking Tau protein (Tau‐KO) and their wild‐type littermates (6‐7 months old; C57BL/6J background) were used in this study divided into stressed and control groups (7 animals per group per genotype)—see also below. Mice were group‐housed under standard environmental conditions (8 am‐8 pm light cycle; 22°C; 55% humidity, ad libitum access to food and water) in accordance with the guidelines for the care and handling of laboratory animals in the Directive 2010/63/EU of the European Parliament and Council. All experiments were conducted in accordance with the Portuguese national authority for animal experimentation, Direção Geral de Alimentação e Veterinária (ID: DGAV9457).

### 2.2 Chronic stress paradigm

Animals were exposed to a 9‐week chronic unpredictable stress (CUS) paradigm during the daily period of light, while control (non‐stressed; CON) mice remained undisturbed in their home cages. The CUS protocol included 4 different stressors: restraint, vibrating platform, overcrowding and exposure to a hot air stream. Animals were exposed to one stressor per day for 3 hours (restraint, vibrating platform, overcrowding) or 30 minutes (hot air stream). The order of stressors and the time of the day at which the stressor was applied were randomly chosen and varied among weeks to promote unpredictability, as previously described.^22^, ^25^, ^28^, ^44^ At the end of the CUS protocol, mice body weight was measured and blood was collected from all animals. Blood serum was isolated after centrifugation and corticosterone (CORT) levels were measured using a radioimmunoassay kit (R&D Systems) according to the manufacturer's instructions.

### 2.3 BrdU treatment

For assessment of cell proliferation, a set of control and stressed WT and Tau‐KO animals (four animals per group) were injected with 5‐bromo‐2′‐deoxyuridine (BrdU; 50 mg/kg per day) for 3 consecutive days before killing. For cell survival monitoring, another set of control and stressed animals of both genotypes (three animals per group) were injected with BrdU (50 mg/kg per day) for 3 consecutive days, 4 weeks before killing—see also Figure 1A.

### 2.4 Tissue preparation

At the end of the CUS protocol, animals were deeply anesthetized (ketamine hydrochloride [150 mg/kg] plus medetomidine [0.3 mg/kg]) and transcardially perfused with saline followed by ice‐cold 4% paraformaldehyde perfusion. Brains were removed, post‐fixed in 4% paraformaldehyde for 2 hours and then transferred to a 30% sucrose solution until they sunk. Then, brains were included in optimal cutting temperature compound (OCT; Tissue Tek, Sakura FineTek), snap‐frozen in liquid nitrogen with 2‐methylbutane and sectioned in a cryostat (Leica CM1900) into 20 μm sections.

### 2.5 Immunofluorescence staining

Coronal brain sections of SVZ and OB were double‐stained for BrdU (1:200; Abcam) followed by doublecortin (DCX; for neuroblast; 1:250; Santa Cruz Biotechnology) or GFAP (for neural‐stem cells; 1:200; Dako) or Olig2 (for oligodendrocytes progenitor cells; 1:300; Merck Millipore) or NeuN (for mature neurons; 1:100; Cell Signalling, Leiden, The Netherlands) for 1 or 2 overnights. Briefly, sections were first washed in PBS (RT) for 3 minutes. Then the sections were heated for 15 minutes in the microwave in citrate buffer (#C9999; Sigma Aldrich/Merck). After washing in PBS, the sections were incubated in HCl 2 mol/L for 30 minutes. After washing in PBS, cells were permeabilized using PBS‐Triton X‐100 0.5% (v/v) for 15 minutes and then incubated on blocking solution (10% foetal bovine serum (v/v) and PBS‐Triton X‐100 0.5% (v/v) at RT for 30 minutes. After incubation with primary antibodies for 1 or 2 overnights (4°C) and PBS washes, sections were incubated with the appropriate secondary antibodies for 2 hours (RT): Alexa Fluor 488 goat anti‐rat for BrdU, 1:1000; Alexa Fluor 568 donkey anti‐goat for DCX, 1:1000; Alexa Fluor 594 donkey anti‐rabbit for GFAP, Olig2 and NeuN, 1:1000; Thermo Fisher Scientific). Cell nuclei were stained with 4′,6‐diamidino‐2‐phenylindole (1:1000; Sigma Aldrich), and slides were mounted with PermaFluor Aqueous Mounting Medium (TA‐006‐FM; Lab Vision, Thermo Fisher Scientific). For cell quantification, confocal images (×40) of SVZ and OB were obtained by the confocal microscope Olympus FluoView FV1000 (Olympus). SVZ and OB images were analysed using the Olympus FluoViewTM FV1000 software. Additionally, for the SVZ analysis, images of the dorsolateral and ventral divisions of posterior sections were used.^9^ For the OB, we used images of three major areas, namely granular cell layer (GCL), mitral cell layer (MCL) and glomerular cell layer (GL) and their correspondence areas were measured with the ImageJ software (http://rsb.info.nih.gov/ij/).

### 2.6 Behavioural testing

#### 2.6.1 Open field

We used an open‐field square arena (43.2 cm × 43.2 cm) surrounded by tall Perspex walls (Med Associates Inc). Each mouse was placed in the centre and allowed to explore the arena for 10 minutes. Infrared beams and manufacturer's software were used to automatically register animals' movements.

#### 2.6.2 Ultrasonic vocalizations

Measurement of ultrasonic vocalizations (USVs) was performed as previously described with some modifications.^45^ Briefly, each animal was placed in a cage for 24 hours. Then, the animal was in close proximity with a female animal, and USVs were recorded for 15 minutes using the Avisoft‐Recorder (version 5.1.04) and manually analysed with AvisoftSAS Lab Pro (version 5.1.22, Avisoft Bioacoustics).

#### 2.6.3 Novel object recognition test

The test arena consisted of a white rectangular box (33 cm × 33 cm × 33 cm). Mice were placed for 20 minutes during 3 consecutive days inside the test arena (habituation). On the following day, mice were placed in the test arena, which contained two identical objects equally distant, and returned to their home cage after 10 minutes of exploration. The next day, the animals were presented a novel object (NO) and one old, familiar object (FO) for 10 minutes; both objects were generally similar regarding height and volume but they were different in shape, colour and texture. Animal's behaviour was recorded, and the time spent exploring each object was manually analysed by the Kinoscope software (http://sourceforge.net/projects/kinoscope/) by an experimenter blind to the animal group. The preference index % (PI%) was calculated based on the formula PI% = time in NO *100/(time in NO + time in FO).

### 2.7 Statistical analysis

Data were analysed using two‐way analysis of variance (ANOVA) before the application of appropriate post hoc pair‐wise comparisons (GraphPad Prism v.6.01; GraphPad Software). Differences were considered statistically significant when *P* <.05. Results are presented as mean ± SEM

## 3 RESULTS

### 3.1 Exposure to chronic stress reduces proliferating cells in the subventricular zone of the adult brain while Tau ablation blocks this stress effect

For clarifying the impact of prolonged stress exposure on the neurogenic niche of the subventricular zone (SVZ) ‐ olfactory bulb (OB) system and monitoring the potential role of Tau in the stress‐driven regulation of cytogenesis, we exposed wild‐type (WT) mice and their littermates lacking Tau protein (Tau‐KO) to a chronic unpredictable stress (CUS) paradigm for 9 weeks (see Figure 1). For detection of newly generated cells in the SVZ‐OB system, we followed the widely used approach of administration of the synthetic nucleotide bromodeoxyuridine (BrdU), which is incorporated into the newly synthesized DNA during the S phase of the cell cycle. To evaluate proliferation in the SVZ, animals were injected with BrdU for three consecutive days before killing (Figure 1A,B). Analysis of BrdU by immunofluorescent staining in the SVZ showed an interaction between *Stress* and *Genotype* in the number of BrdU‐positive cells (two‐way ANOVA, *F*_1,84_ = 4.129, *P* = .045) (Figure 1D,E). Post hoc analysis revealed a significant reduction in the number of BrdU‐positive cells in stressed WT animals when compared to control WTs (*P* = .039) suggesting that chronic stress reduces the proliferation of newly born cells in the SVZ. In contrast, the number of BrdU‐positive cells of stressed Tau‐KO animals was not different from control Tau‐KOs (*P* = .289) (Figure 1E). Additionally, the number of proliferating cells in stressed WT animals was lower when compared with stressed Tau‐KO (*P* < .001) (Figure 1E). No difference was found between WT and Tau‐KO animals under control conditions. Altogether, the above data suggest that, while the absence of Tau does not regulate SVZ proliferation under control conditions, it blocks the reduction of SVZ proliferation under stress.

### 3.2 Chronic stress affects neuroblasts in the SVZ in a Tau‐dependent manner

Neural stem cells (NSCs) in the SVZ may give rise to neuronal and oligodendrocytes precursors (Figure 1C). To monitor the impact of chronic stress on SVZ neuroblasts, brain sections from mice injected with BrdU before sacrifice (Figure 1D) were double‐stained with antibodies against BrdU and DCX; the latter is a cytoskeletal protein expressed in neuroblasts and immature neurons.^46^, ^47^ Quantification of BrdU‐labelled cells that were co‐stained with DCX (Figure 1F—see also Figure S1), revealed a *Stress* × *Genotype* interaction in the percentage of DCX/BrdU double‐labelled cells in the SVZ (two‐way ANOVA *F*_1, 41_ = 6.865, *P* = .0123). Post hoc analysis showed that exposure to CUS reduced the percentage of DCX/BrdU‐labelled neuroblasts in the SVZ of WT animals when compared to control WTs (*P* = .024). However, this was not true for Tau‐KO animals, where the percentage of DCX/BrdU double‐labelled cells in stressed and control Tau‐KOs did not differ (*P* = .365) (Figure 1F). Furthermore, the percentage of DCX/BrdU double‐labelled cells in stressed WT animals was decreased when compared with stressed Tau‐KO (*P* = .006). Again, we did not detect differences in the percentage of DCX/BrdU‐labelled cells between WT and Tau‐KO animals under control conditions.

**FIGURE 2:**
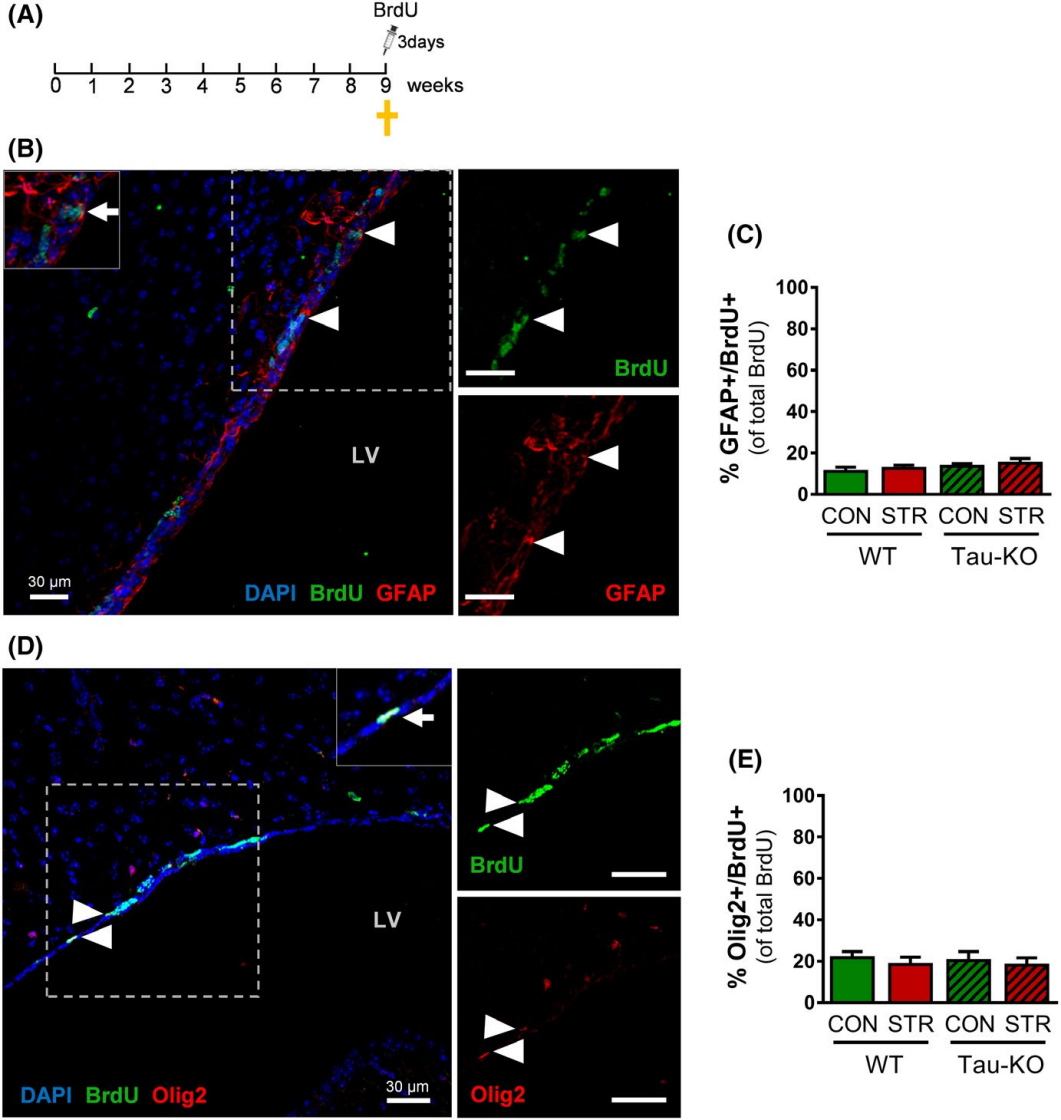
Levels of neural stem cells and oligodendrocytes in the subventricular zone are not altered by chronic stress. A‐E, GFAP/BrdU and Olig2/BrdU immunofluorescent staining of SVZ in animals injected with BrdU before sacrifice. B, Representative microphotograph of BrdU/GFAP double‐labelled cells (arrow head) in the SVZ. C, Chronic stress does not affect the percentage of GFAP/BrdU double‐labelled cells (reflecting neural stem cells) in WT and Tau‐KO animals. D, Olig2/BrdU microphotograph of double‐labelled cells (arrow head) in the SVZ. E, Exposure to chronic stress does not affect the percentage of Olig2/BrdU double‐labelled cells (reflecting oligodendrocytes progenitor cells) in WT and Tau‐KO animals. All numerical data are shown as mean ± SEM; GFAP, glial fibrillary acidic protein; Olig2, oligodendrocyte transcription factor 2; WT, wild type; Tau‐KO, Tau‐knockout

We next monitored the effect of chronic stress and Tau ablation in the pool of NSCs and oligodendrocytes, the two other main cell types present in the SVZ (Figure 1C). To monitor NSCs, we performed staining with antibodies against BrdU and GFAP, a cytoplasmic marker that identifies NSCs in the SVZ (Figure 2A,B—see also Figure S2). As shown in Figure 2C, we found no differences in the percentage of GFAP/BrdU double‐labelled cells in the SVZ among groups, suggesting that chronic stress exposure does not affect this cell population in animals of both genotypes. Furthermore, we have also monitored oligodendrocyte precursors by double labelling with BrdU and Olig2, a nuclear marker that identifies oligodendrocyte progenitors (Figure 2D and Figure S2). Here, we detected no significant differences in Olig2/BrdU‐labelled cells among all groups, indicative of an absence of any significant effect of stress or Tau deletion in this cell population of the SVZ (Figure 2E).

### 3.3 Newly born neurons are differentially regulated by Tau deletion and chronic stress in the different sublayers of the olfactory bulb

**FIGURE 3:**
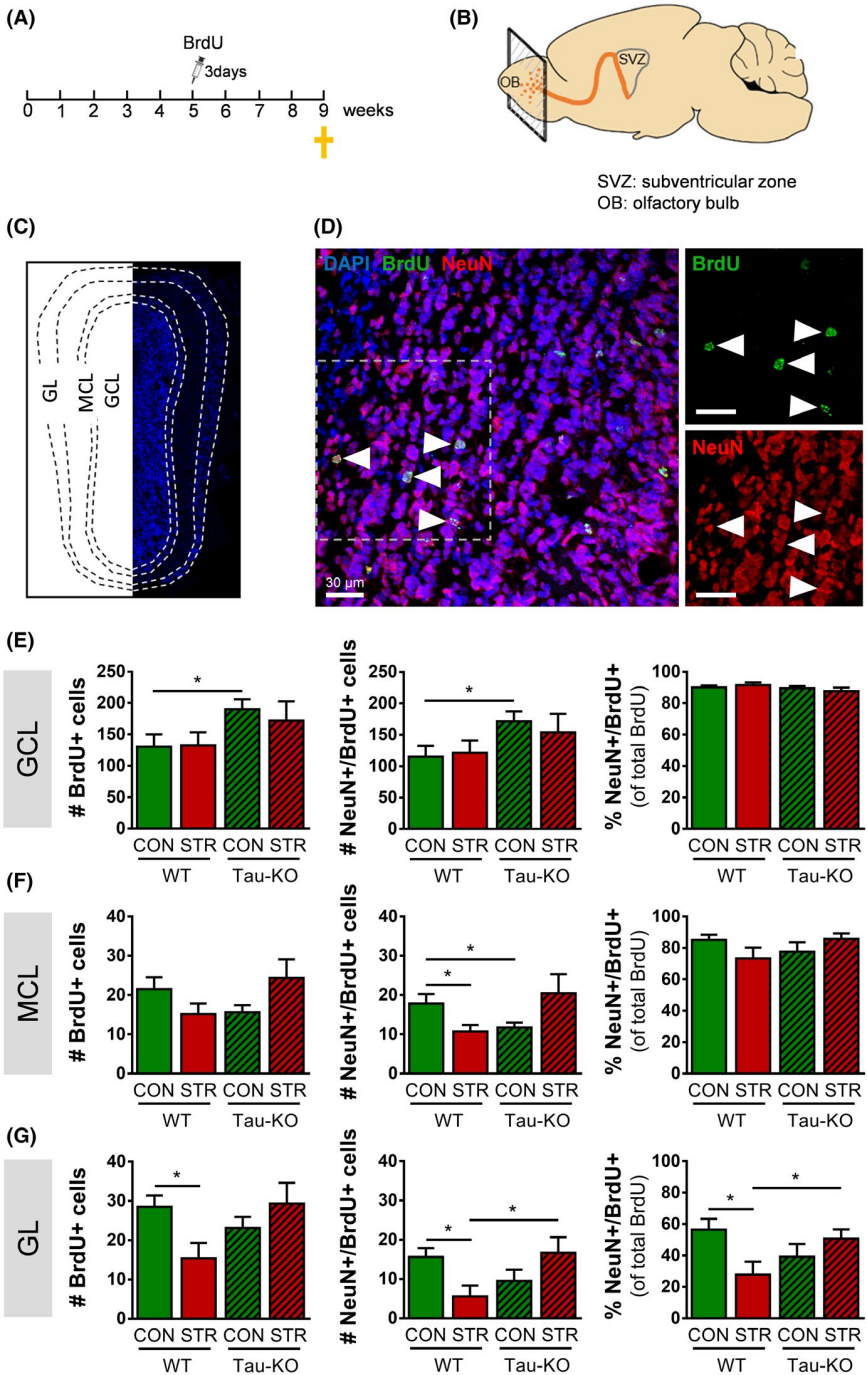
Impact of chronic stress and Tau on newborn neurons in differential sublayers of the olfactory bulb (OB). A, For olfactory bulb (OB) analysis, mice were injected with BrdU 4 weeks before sacrifice. B‐C, Schematic illustration of the mouse brain highlighting the olfactory bulb (OB) level of analysis followed by an olfactory bulb coronal section (C) and the different sublayers analysed; granular cell layer (GCL), mitral cell layer (MCL), glomerular cell layer (GL). D, Representative microphotograph of BrdU/NeuN double‐labelled cells (arrow head) in the OB. E, Tau‐KO animals exhibited increased number of BrdU‐positive cells and NeuN/BrdU double‐labelled cells in the GCL; no differences were found in the percentage of BrdU‐positive cells that are NeuN/BrdU double‐labelled. F, In MCL of OB, Tau‐KO animals exhibited reduced levels of NeuN/BrdU double‐labelled cells compared to WTs and chronic stress reduced NeuN/BrdU cells in WT, but not Tau‐KO, animals. G, In GL, exposure to chronic stress reduced the density of BrdU‐positive cells and NeuN/BrdU double‐labelled cells, as well as, the percentage of NeuN/BrdU cells in GL of WT animals; this stress effect was not found in Tau‐KO animals. All numerical data are shown as mean ± SEM (*P < .05). OB, olfactory bulb; GCL, granular cell layer; MCL, mitral cell layer; GL, glomerular cell layer; BrdU, 5‐bromo‐2′‐deoxyuridine; WT, wild type; Tau‐KO, Tau‐knockout

As the olfactory bulb (OB) is the brain region where the newly born neurons generated within the SVZ migrate, we next analysed the effect of Tau deletion and chronic stress on another set of animals. In this case, animals had been injected with BrdU 4 weeks before the sacrifice, to give enough time for the newly generated cells to reach the OB (Figure 3A). This allows us to monitor the migration of newborn cells and neurons from the SVZ to OB along the different main layers of the OB, namely granular cell layer (GCL), mitral cell layer (MCL) and glomerular cell layer (GL) (Figure 3B,C). For that purpose, we performed double immunofluorescent staining with BrdU and NeuN, a marker that identifies mature neurons (Figure 3D‐ see also Figure S3). Quantification of BrdU cells in the GCL of the OB, the first layer that newborn neurons reach after migrating through the RMS and differentiate into interneurons and specifically into granule cells, showed an overall *Genotype* effect (two‐way ANOVA, *F*_1,30_ = 4.590, *P* = .04), while post hoc analysis revealed a significant increase in BrdU‐labelled cells in control Tau‐KO (*P* = .035) animals when compared with control WT (Figure 3E). No clear impact of stress was detected in WT or Tau‐KO animals. Additionally, quantification of NeuN/BrdU‐labelled cells showed an overall Genotype effect (two‐way ANOVA, F_1,30_ = 4.074, *P* = .05), with post hoc analysis revealing a significant increase in control Tau‐KO (*P* = .035) animals when compared with control WT (Figure 3E). However, the percentage of NeuN/BrdU‐labelled cells among the total BrdU‐labelled cells revealed no statistical differences among groups.

We next monitored the BrdU‐ and NeuN‐positive cells in the MCL of the OB, the second layer of migration and differentiation into granule cells in the OB. Two‐way ANOVA analysis of BrdU‐labelled cells showed a *Stress × Genotype* interaction effect (*F*_1, 30_ = 4.814, *P* = .036). Similarly, quantification of NeuN+/BrdU+ cells showed a *Stress × Genotype* interaction effect (*F*_1, 30_ = 6.565, *P* = .015) accompanied by a decrease in stressed WT animals when compared to control WT (*P* = .037). Additionally, Tau‐KO CONs showed decreased number of NeuN+/BrdU+ cells when compared to WT CONs (*P* = .046). When we calculated the percentage of NeuN/BrdU double‐labelled cells among the total BrdU‐positive cells, we found a similar *Stress × Genotype* interaction effect (*F*_1, 30_ = 4.257, *P* = .047) (Figure 3F).

We also monitored the GL of OB, the last layer of OB and where cells differentiate into periglomerular cells. Quantification of BrdU‐labelled cells in the GL revealed a *Stress × Genotype* interaction effect (two‐way ANOVA, *F*_1,30_ = 6.071, *P* = .0197). Post hoc analysis revealed a significant decrease in BrdU‐labelled cells in stressed WT mice when compared with control WTs (*P* = .014). However, the levels of BrdU‐labelled cells in stressed Tau‐KO animals were not different from the levels of control Tau‐KOs (Figure 3G). Similarly, quantification of NeuN/BrdU‐labelled cells showed a Stress × Genotype interaction (F_1, 30_ = 7.834, *P* = .008). Post hoc analysis showed that exposure to chronic stress reduced the number of NeuN/BrdU‐labelled cells in WT animals (*P* = .012), but not in Tau‐KO when compared to their corresponding controls (Figure 3G). Moreover, in stressed WT animals, these cells were reduced when compared to Tau‐KO stressed (*P* = .048). We next calculated the percentage of NeuN/BrdU double‐labelled cells and found a Stress × Genotype interaction effect (*F*_1, 30_ = 7.476, *P* = .010). Further analysis showed that stressed WT animals presented a reduced percentage of NeuN/BrdU‐double‐labelled cells when compared to control WTs (*P* = .018). However, the percentage of NeuN/BrdU‐labelled cells was not altered in stressed Tau‐KO in comparison with control Tau‐KO (*P* = .265) but was significantly higher when compared to stressed WT (*P* = .036). Altogether, the above data suggest that exposure to chronic stress reduced the newly born neurons found in the MCL and GL of OB, and this decrease was blocked in Tau‐KO animals indicating that Tau protein is critical in stress‐induced neurogenic suppression.

### 3.4 Tau deletion does not interfere with the endocrine response to stress but blocks the related behavioural changes

**FIGURE 4:**
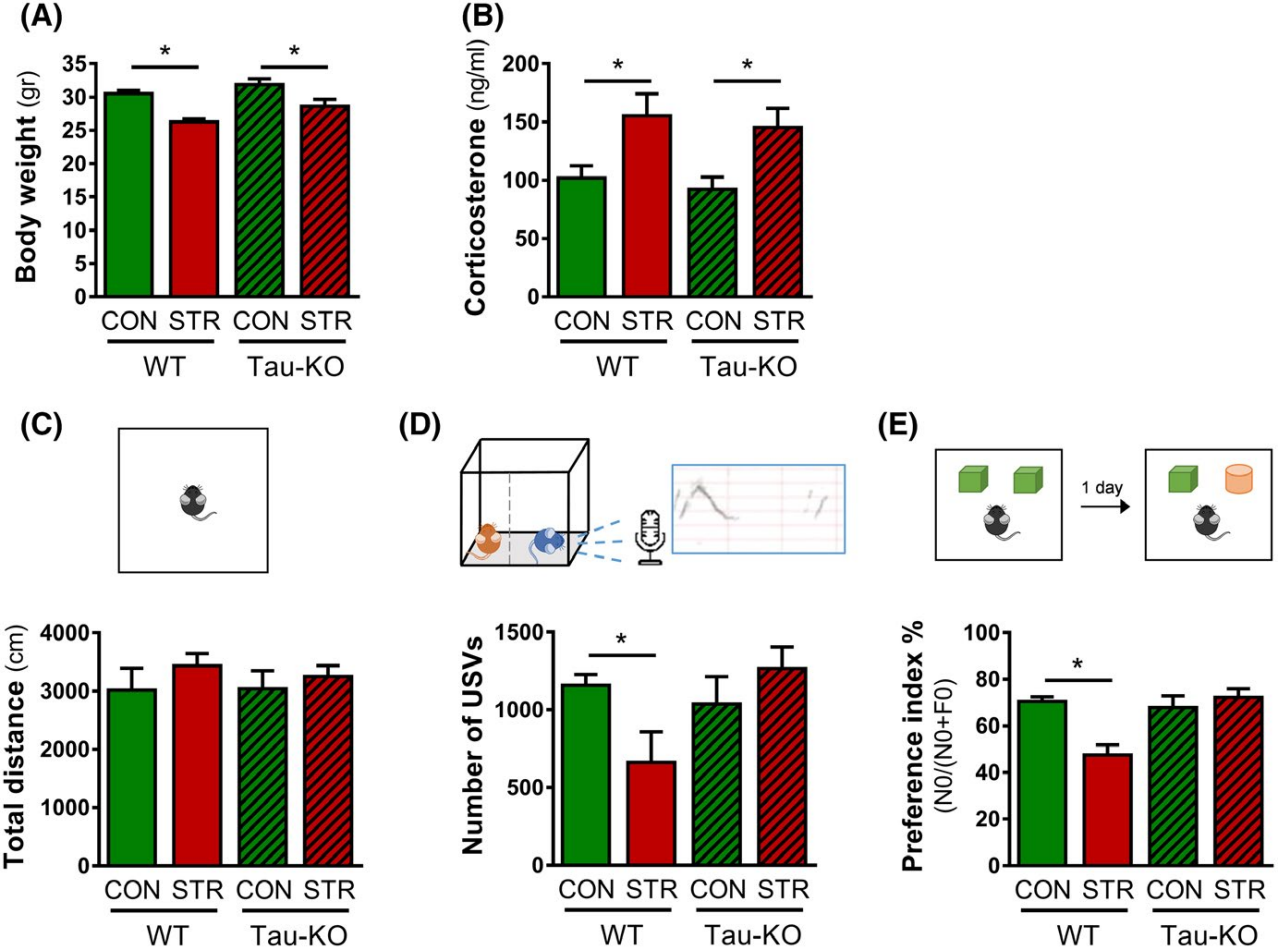
Tau deletion does not interfere with endocrine response to stress but attenuates stress‐induced behavioural impairment. A‐B, Stressed animals of both WT and Tau‐KO genotype exhibited reduced body weight (A) and increased levels of corticosterone, the main stress hormones (B), when compared with their corresponding control animals. C, Total distance travelled in the open‐field arena was not different among groups. D, Chronic stress decreased the number of ultrasonic vocalizations (USVs) in WT animals indicating deficits of emotional status; no effect of stress was found in Tau‐KO animals. E, Preference index in the Novel Object Recognition test was reduced in stressed WTs when compared to WT controls; this stress effect was not found in Tau‐KOs suggesting a Tau‐dependent cognitive impairment caused by chronic stress. All numerical data are shown as mean ± SEM (*P <.05). USVs, ultrasonic vocalizations; WT, wild type; Tau‐KO, Tau‐knockout

Exposure to chronic stress is known to impact organism's homeostasis with stressed animals exhibiting reduced body weight, elevation of stress hormones and behavioural deficits^31^, ^32^, ^48^ For monitoring stress efficacy and impact on behaviour, we performed biometric, biochemical and behavioural analysis. Body weight was measured at the end of the stress period and statistical analysis revealed an overall *Stress* effect (two‐way ANOVA, *F*1_,24_ = 24.92, *P* < .0001) (Figure 4A). Specifically, stressed WT animals exhibited reduced body weight when compared to control WT animals (*P* < .0001). Similar to WTs, stressed Tau‐KO animals exhibited reduced body weight in comparison with control Tau‐KOs (*P* = .03) suggesting that stress affected body weight independently of Tau. Similarly, analysis of the levels of corticosterone, the main stress hormone, showed an overall *Stress* effect (two‐way ANOVA, *F*_1,24_ = 13.48, *P* = .001). Furthermore, stressed animals of both genotypes presented increased levels of blood corticosterone when compared to the corresponding CON group (WT CON vs. WT STR: *P* < .0001 and Tau‐KO CON vs. Tau‐KO STR: *P* = .033) (Figure 4B). These results suggest that the absence of Tau does not interfere with the endocrine response to stress leading to the expected elevation in corticosterone levels. Next, we monitored different behavioural domains starting with locomotion and overall activity of the animals. The Open‐field (OF) test showed no differences in the total distance travelled among groups indicating no changes in locomotion under stress conditions in both WT and Tau‐KO animals (Figure 4C). Additionally, we monitored ultrasonic vocalizations (USVs) of the animals as an index of their emotional status.^45^ Our results showed a *Stress* × *Genotype* interaction in the number of USVs emitted by each animal (two‐way ANOVA *F*_1, 24_ = 5.578, *P* = .0026). Further analysis revealed that stressed WT animals exhibited a reduced number of USVs when compared to WT controls (*P* = .035) (Figure 4D). On the contrary, Tau‐KO animals were not affected by stress as control and stressed Tau‐KO animals showed similar levels of USVs (*P* = .327). Cognitive performance was also monitored by Novel object recognition. We found an interaction between *Stress* and *Genotype* in the preference index (two‐way ANOVA, *F*_1,24_ = 12.23, *P* = .002) (Figure 4E). Post hoc analysis revealed a significant reduction in the preference index in stressed WT animals when compared to control WTs (*P* = .002) while no differences were found between stressed and control Tau‐KO animals (*P* = .854).

## 4 DISCUSSION

Fifty‐five years after the first report of neurogenesis in the adult brain,^49^ the specific role and the mechanisms that regulate the generation of newborn neurons integrated into the adult brain are still to be fully understood. Although the extent and relevance of adult neurogenesis in humans are currently debated,^15^, ^16^ accumulating evidence supports the involvement of neurogenesis in brain morphofunctional response to different stimuli (eg exercise, environmental enrichment vs. sleep deprivation, environmental stress) and brain pathologies (depression, post‐traumatic stress disorder, Alzheimer's disease).^28^, ^50^, ^51^ Furthermore, the possibility to induce neural precursors to generate new neurons is an attractive prospect for neuro‐replacement therapy in different pathological conditions characterized by neuronal loss (e.g. Alzheimer's disease, brain trauma). The role of SVZ neurogenesis in the adult brain function and pathology has been less investigated compared with hippocampal neurogenesis despite that the SVZ exhibits the highest number of proliferative cells in the adult brain.^4^, ^5^ Moreover, besides the OB (in rodents^52^, ^53^; or the striatum (in humans^54^), other neocortical regions after stroke or trauma are shown to receive newly born cells/neurons generated in the SVZ.^40^, ^55^, ^56^

**FIGURE 5:**
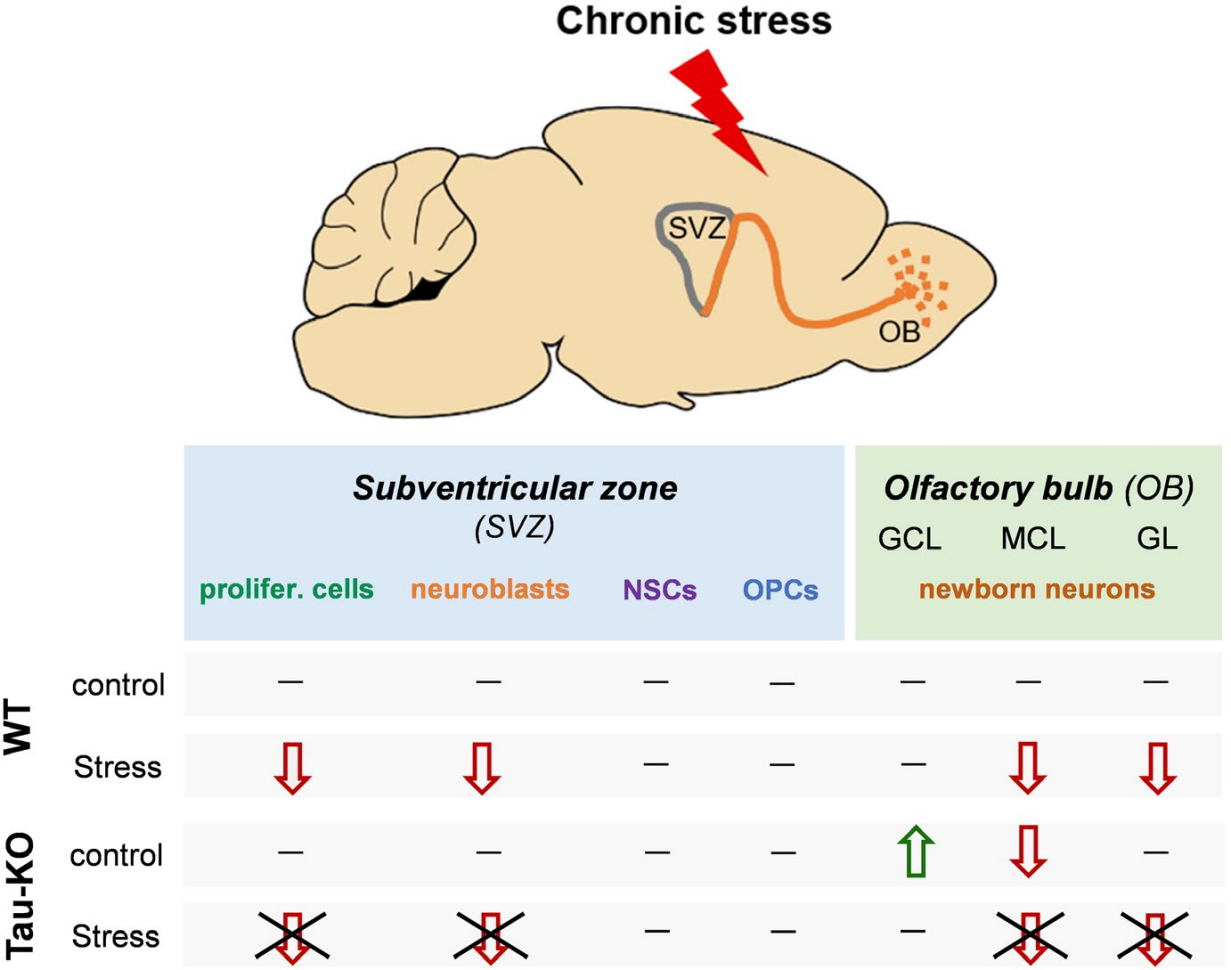
Summary of the impact of chronic stress on different cell populations in the subventricular zone ‐ olfactory bulb neurogenic niche. Chronic stress suppresses proliferation, neuronal, but not oligodendrocytic, differentiation and maturation of newly born cells in the subventricular zone (SVZ) and olfactory bulb (OB) of the adult brain as assessed by decreased levels of proliferating cells and neuroblasts in SVZ and reduced newborn neurons in the mitral cell and glomerular cell lB neurogenic niche of the adult brainayers (MCL and GL, respectively) of OB. However, the stress impact on the above cell populations was blocked in animals lacking Tau (Tau‐KO). These findings suggest that Tau protein is essentially involved in the neurogenesis‐suppressing role of chronic stress on the SVZ‐OB neurogenic niche of the adult brain in line with previously reported reduction of the hippocampal neurogenesis by stress.^22^, ^33^ Interestingly, compared to wild type (WT), newborn neurons of Tau‐KO animals seem to be accumulated in the first layer of the OB, the granular cell layer (GCL), indicating delayed migration of newborn neurons in the other OB cell layers and subsequently, decreased number of these cells in the mitral cell layer (MCL). GCL, granular cell layer; MCL, mitral cell layer; GL, glomerular cell layer; NSCs, neural stem cells; OPCs, oligodendrocytes progenitor cells; WT; wild‐type, Tau‐KO, Tau‐knockout

The current study focused on the analysis of neurogenesis in the SVZ‐OB system of the adult rodent brain after prolonged exposure to environmental stress. Clinical and experimental evidence has long shown that exposure to stressful conditions is a strong precipitant of depressive pathology while a cardinal feature of the response to chronic stress is the atrophy of specific brain regions, as detected by both brain imaging and stereological techniques.^57^, ^58^ These plastic changes of the brain include dendritic atrophy and synaptic loss accompanied by the suppressed generation of newly born cells in specific areas of the adult brain.^28^, ^29^, ^48^, ^57^, ^59^ Whereas the hippocampus has been the main focus of a plethora of clinical and experimental studies of depressed and/or stress‐exposed human subjects and related animal models, clinical studies have also reported that adults with a history of early life stress or major depressive disorder present reduced OB volume and odorant detection impairment ^60^, ^61^, ^62^, ^63^ indicating the potential impact of chronic stress on the OB. In line with that clinical evidence, few experimental studies on rodents exposed to chronic stress and/or to high levels of stress hormones, glucocorticoids, have shown reduced SVZ neurogenesis and olfactory deficits along with depressive‐like and anxiety symptoms.^43^, ^64^, ^65^ Different stress paradigms, such as maternal separation, repeated exposure to forced swim stress and chronic administration of corticosterone resulted in reduced BrdU‐labelled proliferating cells in the SVZ.^43^, ^64^, ^65^, ^66^ In line with these reports, we hereby demonstrate that 9‐week exposure to a CUS protocol reduced both proliferation and neuronal differentiation of newly born cells in the SVZ, as assessed by the reduced number of BrdU‐labelled cells as well as DCX/BrdU‐labelled neuronal precursors and immature neurons in SVZ (Figure 5). On the other hand, exposure to short stress periods (e.g. 2‐day repeated foot shock stress paradigm ^67^) or a milder stress paradigm (e.g. chronic mild stress ^42^ or corticosterone ^64^) do not seem to impact SVZ neurogenesis; nevertheless, the above stress paradigms reduced neurogenesis in the hippocampus indicating a differential vulnerability of proliferating cells to stress and stress hormones between the dentate gyrus (DG) and SVZ neurogenic niches (see also ^64^). This is of great importance as different areas of the adult brain are shown to exhibit different vulnerability to the detrimental effects of chronic stress on their plasticity and function (e.g. hippocampus vs. frontal cortex ^68^) while other brain areas respond with opposite effects to chronic stress; for instance, stress causes atrophy to adult hippocampus whereas hypertrophy to amygdala and nucleus accumbens.^48^, ^69^

Following a 4‐week period of newly born cells survival and migration into the OB, we found that the 9‐week CUS protocol also suppress the BrdU‐labelled cell population in the MCL and GL of the OB; This was accompanied by a reduced number of NeuN/BrdU‐labelled newborn neurons in OB of the adult brain (Figure 5). These findings indicate that chronic stress diminishes neuronal maturation as well as the survival rate of newly born neurons in the OB that may contribute to the previously described deficits of olfactory memory induced by prolong stress and/or corticosterone exposure.^43^, ^64^ The current findings on both SVZ and OB brain areas suggest that 9 weeks of chronic stress exposure suppress proliferation, neuronal differentiation and maturation of newly born cells in the SVZ and OB, respectively (Figure 5). Together with previous studies showing that stress triggers neuronal atrophy and synaptic loss in pre‐existing (older) hippocampal and cortical neurons,^48^, ^70^ as well as suppresses the genesis of newborn neurons in the hippocampal dentate gyrus,^22^, ^23^, ^48^, ^71^ the current study provides solid evidence about the participation of the SVZ‐OB neurogenic system in the network of brain areas damaged by chronic stress. Through an integrated manner, chronic stress damages various domains of behavioural performance such as different types of memory (i.e. associative, spatial and odour memories) and emotional status (e.g. anxiety levels, depressive behaviour).

Tau is an important protein involved in the regulation of cytoskeletal assembly and different cellular processes, such as axonal branching and transport, as well as in neuronal polarity, migration and differentiation.^22^, ^72^, ^73^, ^74^ Despite the compensatory mechanisms (e.g. increased expression of other cytoskeletal proteins) that have been suggested to attribute to the lack of gross behavioural and neurostructural abnormalities in animals that lack Tau protein (Tau‐KO), the absence of Tau appears to cause a transient delay in the dendritic maturation of new‐born neurons ^33^ as well as a delay in their migration.^73^, ^75^ Moreover, it was recently shown that 14‐month‐old Tau‐KO mice exhibit increased BrdU‐labelled proliferating cells in the SVZ.^76^ In contrast, our analysis of BrdU‐labelled proliferating cell in SVZ did not detect a significant difference between WT and Tau‐KO animals; this difference may be attributed to the age difference between our (6‐7‐month‐old mice) and Criado‐Marrero study (14 months old).^76^ However, our findings demonstrate a significant increase in the number of newborn neurons in the OB, a region that was not monitored in Criado‐Marrero's study.^76^ Moreover, this increase in the number of newborn neurons was detected in the first layer of the OB (granular cell layer; GCL) of Tau‐KO animals accompanied by a tendency for a decrease in the next OB layers (mitral and glomerular cell layers—see Figure 3 and Suppl. Figure 3), indicative of a delay of neuronal migration in OB led by the absence of Tau. Although the precise mechanisms through which lack of Tau may induce neuronal migration deficits in the OB are still under investigation, inhibition of the Rho‐ROCK signalling pathway by Tau absence in glioblastoma cells was recently shown to induce the remodelling of the actin cytoskeleton leading to delayed cell migration.^77^ Alternatively, the interplay between Tau protein and the transduction of reelin, a protein that is crucial in neuronal migration and to the formation of synaptic connections in the brain, may have an equally important role here.^78^, ^79^, ^80^ Moreover, the malfunction of Tau is causally related to cytoskeletal dysregulation, neuronal malfunction and atrophy under different pathological conditions including AD as well as stroke and brain trauma.^48^, ^70^, ^81^, ^82^, ^83^ As SVZ neurogenesis is suggested to participate in the endogenous regenerative response of the brain to stroke or trauma,^40^, ^55^, ^56^ future studies should clarify the potential role of Tau in the adult neurogenic process under these pathological conditions, too.

Recent animal studies from our team and others have proposed the involvement of Tau in the regulation of adult neurogenesis of the hippocampus after exposure to acute or prolonged stress.^22^, ^33^ Specifically, exposure to stress leads to Tau hyperphosphorylation and accumulation in newly born neurons of the adult brain.^2^ It is known that dephosphorylated Tau binds more stable to microtubules while increased phosphorylation of Tau is shown to reduce its microtubule‐binding capacity leading to microtubule instability.^84^ Through a constant regulation of its phosphorylation‐dephosphorylation equilibrium, Tau protein is involved in many cellular functions as it regulates the cytoskeletal stability influencing morphogenesis of neurons.^85^ Thus, stress‐evoked alterations of the tight control of Tau phosphorylation could impair the complex and tight regulation of Tau, diminishing the cellular control over the cytoskeletal dynamics and network essential for proliferating cells and neuroblasts. Interestingly, animals lacking Tau were spared from the neurogenesis‐damaging effects of chronic stress as, in contrast to WTs, stressed Tau‐KO presented no reduction of proliferating cells and neuroblasts in the SVZ, followed by lack of decreased newborn neurons in the OB (Figure 5). Extending previous evidence about Tau‐dependent suppression of the hippocampal neurogenesis under stress conditions,^22^, ^33^ the current study suggests the essential mediation of Tau in the stress‐driven neurogenic deficits in the SVZ‐OB system of the adult brain. Moreover, progenitor cells in the hippocampus and SVZ are also able to differentiate into non‐neuronal cell types (e.g. astrocytes, oligodendrocytes); however, our knowledge about whether and how chronic stress impacts other types of newborn cells (non‐neuronal ones) in the adult brain remains limited. Our findings suggest that the population of oligodendrocyte progenitor cells in the SVZ is not affected by stress indicating that the detrimental impact of chronic stress on the SVZ‐OB niche is mainly neuronal with an essential mediating role for Tau protein. Indeed, the neuroprotective role of Tau reduction against chronic stress is extended beyond newly born cells (and neurogenesis) as it is also evident in old, pre‐existing neurons of the hippocampus as well as of other brain areas (e.g. prefrontal cortex).^48^, ^70^ Moreover, emerging evidence from animal models of diverse brain pathologies (e.g. Alzheimer's disease, epilepsy, stroke, traumatic brain injury) ^81^, ^83^, ^86^ suggests Tau as a converging protein of neuronal damage between different insults and disorders highlighting its broad neuroplastic and neuropathological role.^85^

In summary, the current study provides novel insights about the involvement of Tau protein in the mechanisms that reduce cell proliferation, neuronal differentiation and migration within the SVZ‐OB neurogenic niche under prolonged stressful conditions. Together with previous work suggesting the role of Tau in stress‐evoked hippocampal plasticity changes,^22^, ^33^, ^48^ these findings bring further information about the biological underpinnings of the stress‐driven deficits on the adult brain circuits regulating mood and cognition. A better understanding of the mechanisms underlying the neuroplastic effect of chronic stress on the adult brain may help the development of targeted therapies for stress‐related disorders, such as depression and Alzheimer's disease, which are characterized by deficits of neuronal plasticity.

## CONFLICT OF INTEREST

The authors declare no conflict of interest.

## AUTHOR CONTRIBUTIONS

CD was involved in all stages of the experimental procedures, data collection and analysis, interpretation and manuscript preparation. PP, CM, MM, and LGP have contributed to immunohistochemical staining and confocal microscope analysis and involved in the preparation of the manuscript. SV and JMB provide materials and critically reviewed the manuscript. LP and IS supervised these studies and were involved in interpretation and manuscript preparation.

## Supporting information

Fig S1‐S3

## Data Availability

The data that support the findings of this study are available from the corresponding author upon reasonable request.

## References

[1] Sailor KA , Schinder AF , Lledo PM . Adult neurogenesis beyond the niche: its potential for driving brain plasticity. Curr Opin Neurobiol. 2017;42:111‐117. 10.1016/j.conb.2016.12.001 28040643

[2] La Rosa C , Parolisi R , Bonfanti L . Brain structural plasticity: from adult neurogenesis to immature neurons. Front Neurosci. 2020;14:75. 10.3389/fnins.2020.00075 32116519 PMC7010851

[3] Ming GL , Song H . Adult neurogenesis in the mammalian brain: significant answers and significant questions. Neuron. 2011;70:687‐702. 10.1016/j.neuron.2011.05.001 21609825 PMC3106107

[4] Alvarez‐Buylla A , García‐Verdugo JM . Neurogenesis in adult subventricular zone. J Neurosci. 2002;22:629‐634. 10.1523/jneurosci.22-03-00629.2002 11826091 PMC6758521

[5] Hong XP , Peng CX , Wei W , et al. Relationship of adult neurogenesis with tau phosphorylation and GSK‐3β activity in subventricular zone. Neurochem Res. 2011;36:288‐296. 10.1007/s11064-010-0316-y 21061060

[6] Alonso M , Lepousez G , Wagner S , et al. Activation of adult‐born neurons facilitates learning and memory. Nat Neurosci. 2012;15:897‐904. 10.1038/nn.3108 22581183

[7] Rochefort C , Gheusi G , Vincent JD , Lledo PM . Enriched odor exposure increases the number of newborn neurons in the adult olfactory bulb and improves odor memory. J Neurosci. 2002;22:2679‐2689. 10.1523/jneurosci.22-07-02679.2002 11923433 PMC6758329

[8] Nora Abrous D , Koehl M , LE Moal M . Adult neurogenesis: from precursors to network and physiology I. Neurogenesis in the adult brain: a new paradigm for structure‐ function relationships. Physiol Rev. 2005;85:523‐569. 10.1152/physrev.00055.2003 15788705

[9] Falcão AM , Palha JA , Ferreira AC , Marques F , Sousa N , Sousa JC . Topographical analysis of the subependymal zone neurogenic niche. PLoS ONE. 2012;7:e38647. 10.1371/journal.pone.0038647 22745673 PMC3379980

[10] Ming G , Song H . Adult neurogenesis in the mammalian central nervous system. Annu Rev Neurosci. 2005;28:223‐250. 10.1146/annurev.neuro.28.051804.101459 16022595

[11] Lledo PM , Valley M . Adult olfactory bulb neurogenesis. Cold Spring Harb Perspect Biol. 2016;8:a018945. 10.1101/cshperspect.a018945 27235474 PMC4968158

[12] Menn B , Garcia‐Verdugo JM , Yaschine C , Gonzalez‐Perez O , Rowitch D , Alvarez‐Buylla A . Origin of oligodendrocytes in the subventricular zone of the adult brain. J Neurosci. 2006;26:7907‐7918. 10.1523/JNEUROSCI.1299-06.2006 16870736 PMC6674207

[13] Lim DA , Alvarez‐Buylla A . The adult ventricular–subventricular zone (V‐SVZ) and olfactory bulb (OB) neurogenesis. Cold Spring Harb Perspect Biol. 2016;8:a018820. 10.1101/cshperspect.a018820 27048191 PMC4852803

[14] Kempermann G , Gage FH , Aigner L , et al. Human adult neurogenesis: evidence and remaining questions. Cell Stem Cell. 2018;23:25‐30. 10.1016/j.stem.2018.04.004 29681514 PMC6035081

[15] Sorrells SF , Paredes MF , Cebrian‐Silla A , et al. Human hippocampal neurogenesis drops sharply in children to undetectable levels in adults. Nature. 2018;555:377‐381. 10.1038/nature25975 29513649 PMC6179355

[16] Moreno‐Jiménez EP , Flor‐García M , Terreros‐Roncal J , et al. Adult hippocampal neurogenesis is abundant in neurologically healthy subjects and drops sharply in patients with Alzheimer's disease. Nat Med. 2019;25:554‐560. 10.1038/s41591-019-0375-9 30911133

[17] Marlatt MW , Potter MC , Bayer TA , van Praag H , Lucassen PJ . Prolonged running, not fluoxetine treatment, increases neurogenesis, but does not alter neuropathology, in the 3xTg mouse model of Alzheimer's disease. Curr Top Behav Neurosci. 2013;15:313‐340. 10.1007/7854_2012_237 23670818 PMC4554490

[18] Rodríguez JJ , Jones VC , Tabuchi M , et al. Impaired adult neurogenesis in the dentate gyrus of a triple transgenic mouse model of Alzheimer's disease. PLoS ONE. 2008;3:e2935. 10.1371/journal.pone.0002935 18698410 PMC2492828

[19] Hamilton LK , Aumont A , Julien C , Vadnais A , Calon F , Fernandes KJL . Widespread deficits in adult neurogenesis precede plaque and tangle formation in the 3xTg mouse model of Alzheimer's disease. Eur J Neurosci. 2010;32:905‐920. 10.1111/j.1460-9568.2010.07379.x 20726889

[20] Valero J , Bernardino L , Cardoso FL , et al. Impact of neuroinflammation on hippocampal neurogenesis: relevance to aging and Alzheimer's disease. J Alzheimer's Dis. 2017;60:S161‐S168. 10.3233/JAD-170239 28671124

[21] Boldrini M , Santiago AN , Hen R , et al. Hippocampal granule neuron number and dentate gyrus volume in antidepressant‐treated and untreated major depression. Neuropsychopharmacology. 2013;38:1068‐1077. 10.1038/npp.2013.5 23303074 PMC3629406

[22] Dioli C , Patrício P , Trindade R , et al. Tau‐dependent suppression of adult neurogenesis in the stressed hippocampus. Mol Psychiatry. 2017;22:1110‐1118. 10.1038/mp.2017.103 28555078

[23] Alves ND , Patrício P , Correia JS , et al. Chronic stress targets adult neurogenesis preferentially in the suprapyramidal blade of the rat dorsal dentate gyrus. Brain Struct Funct. 2018;223:415‐428. 10.1007/s00429-017-1490-3 28852856

[24] Sotiropoulos I , Catania C , Riedemann T , et al. Glucocorticoids trigger Alzheimer disease‐like pathobiochemistry in rat neuronal cells expressing human tau. J Neurochem. 2008;107:385‐397. 10.1111/j.1471-4159.2008.05613.x 18691381

[25] Sotiropoulos C , Catania C , Pinto LG , et al. Stress acts cumulatively to precipitate Alzheimer's disease‐like tau pathology and cognitive deficits. J Neurosci. 2011;31:7840‐7847. 10.1523/JNEUROSCI.0730-11.2011 21613497 PMC6633145

[26] Baglietto‐Vargas D , Chen Y , Suh D , et al. Short‐term modern life‐like stress exacerbates Aβ‐pathology and synapse loss in 3xTg‐AD mice. J Neurochem. 2015;134:915‐926. 10.1111/jnc.13195 26077803 PMC4792118

[27] Khan AR , Geiger L , Wiborg O , Czéh B . Stress‐induced morphological, cellular and molecular changes in the brain—lessons learned from the chronic mild stress model of depression. Cells. 2020;9:1026. 10.3390/cells9041026 PMC7226496 32326205

[28] Dioli C , Patrício P , Sousa N , et al. Chronic stress triggers divergent dendritic alterations in immature neurons of the adult hippocampus, depending on their ultimate terminal fields. Transl Psychiatry. 2019;9(1):143. 10.1038/s41398-019-0477-7 31028242 PMC6486609

[29] Bessa JM , Ferreira D , Melo I , et al. The mood‐improving actions of antidepressants do not depend on neurogenesis but are associated with neuronal remodeling. Mol Psychiatry. 2009;14:764‐773. 10.1038/mp.2008.119 18982002

[30] Chen CC , Huang CC , Sen HK . Chronic social stress affects synaptic maturation of newly generated neurons in the adult mouse dentate gyrus. Int J Neuropsychopharmacol. 2015;19:pyv097. 10.1093/ijnp/pyv097 26346341 PMC4815468

[31] Van Bokhoven P , Oomen CA , Hoogendijk WJG , Smit AB , Lucassen PJ , Spijker S . Reduction in hippocampal neurogenesis after social defeat is long‐lasting and responsive to late antidepressant treatment. Eur J Neurosci. 2011;33:1833‐1840. 10.1111/j.1460-9568.2011.07668.x 21488984

[32] Touma C . Stress and affective disorders: animal models elucidating the molecular basis of neuroendocrine‐behavior interactions. Pharmacopsychiatry. 2011;44:S15‐S26. 10.1055/s-0031-1271702 21544741

[33] Pallas‐Bazarra N , Jurado‐Arjona J , Navarrete M , et al. Novel function of Tau in regulating the effects of external stimuli on adult hippocampal neurogenesis. EMBO J. 2016;35:1417‐1436. 10.15252/embj.201593518 27198172 PMC4876034

[34] Breuzard G , Hubert P , Nouar R , et al. Molecular mechanisms of Tau binding to microtubules and its role in microtubule dynamics in live cells. J Cell Sci. 2013;126:2810‐2819. 10.1242/jcs.120832 23659998

[35] Cabrales Fontela Y , Kadavath H , Biernat J , Riedel D , Mandelkow E , Zweckstetter M . Multivalent cross‐linking of actin filaments and microtubules through the microtubule‐associated protein Tau. Nat Commun. 2017;8(1):1981. 10.1038/s41467-017-02230-8 29215007 PMC5719408

[36] Elie A , Prezel E , Guérin C , et al. Tau co‐organizes dynamic microtubule and actin networks. Sci Rep. 2015;5:9964. 10.1038/srep09964 25944224 PMC4421749

[37] Farias GA , Muñoz JP , Garrido J , Maccioni RB . Tubulin, actin, and tau protein interactions and the study of their macromolecular assemblies. J Cell Biochem. 2002;85:315‐324. 11948687 10.1002/jcb.10133

[38] He HJ , Wang XS , Pan R , Wang DL , Liu MN , He RQ . The proline‐rich domain of tau plays a role in interactions with actin. BMC Cell Biol. 2009;10:81. 10.1186/1471-2121-10-81 19895707 PMC2784441

[39] Gould E , Reeves AJ , Graziano MSA , Gross CG . Neurogenesis in the neocortex of adult primates. Science (80‐ ). 1999;286:548‐552. 10.1126/science.286.5439.548 10521353

[40] Brandt MD , Storch A . Neurogenesis in the adult brain: from bench to bedside? Fortschritte der Neurol Psychiatr. 2008;76:517‐529. 10.1055/s-2008-1038218 18712664

[41] Ming GL , Song H . Adult neurogenesis in the mammalian brain: significant answers and significant questions. Neuron. 2011;70:687‐702. 10.1016/j.neuron.2011.05.001 21609825 PMC3106107

[42] Surget A , Saxe M , Leman S , et al. Drug‐dependent requirement of hippocampal neurogenesis in a model of depression and of antidepressant reversal. Biol Psychiatry. 2008;64:293‐301. 10.1016/j.biopsych.2008.02.022 18406399

[43] Hitoshi S , Maruta N , Higashi M , Kumar A , Kato N , Ikenaka K . Antidepressant drugs reverse the loss of adult neural stem cells following chronic stress. J Neurosci Res. 2007;85:3574‐3585. 10.1002/jnr.21455 17668856

[44] Cerqueira JJ , Mailliet F , Almeida OFX , Jay TM , Sousa N . The prefrontal cortex as a key target of the maladaptive response to stress. J Neurosci. 2007;27:2781‐2787. 10.1523/JNEUROSCI.4372-06.2007 17360899 PMC6672565

[45] Scattoni ML , Ricceri L , Crawley JN . Unusual repertoire of vocalizations in adult BTBR T+tf/J mice during three types of social encounters. Genes, Brain Behav. 2011;10:44‐56. 10.1111/j.1601-183X.2010.00623.x 20618443 PMC2972364

[46] Francis F , Koulakoff A , Boucher D , et al. Doublecortin is a developmentally regulated, microtubule‐associated protein expressed in migrating and differentiating neurons. Neuron. 1999;23:247‐256. 10.1016/S0896-6273(00)80777-1 10399932

[47] Horesh D , Sapir T , Francis F , et al. Doublecortin, a stabilizer of microtubules. Hum Mol Genet. 1999;8:1599‐1610. 10.1093/hmg/8.9.1599 10441322

[48] Lopes S , Vaz‐Silva J , Pinto V , et al. Tau protein is essential for stress‐induced brain pathology. Proc Natl Acad Sci. 2016;113:E3755‐E3763. 10.1073/pnas.1600953113 27274066 PMC4932951

[49] Altman J , Das GD . Autoradiographic and histological evidence of postnatal hippocampal neurogenesis in rats. J Comp Neurol. 1965;124:319‐335. 10.1002/cne.901240303 5861717

[50] Mu Y , Gage FH . Adult hippocampal neurogenesis and its role in Alzheimer's disease. Mol Neurodegener. 2011;6:85. 10.1186/1750-1326-6-85 22192775 PMC3261815

[51] Lucassen PJ , Meerlo P , Naylor AS , et al. Regulation of adult neurogenesis by stress, sleep disruption, exercise and inflammation: Implications for depression and antidepressant action. Eur Neuropsychopharmacol. 2010;20:1‐17. 10.1016/j.euroneuro.2009.08.003 19748235

[52] Doetsch F , Caille I , Lim DA , Garcia‐Verdugo JM , Alvarez‐Buylla A . Subventricular zone astrocytes are neural stem cells in the adult mammalian brain. Cell. 1999;97:703‐716. 10.1016/S0092-8674(00)80783-7 10380923

[53] Eiriz MF , Valero J , Malva JO , Bernardino L . New insights into the role of histamine in subventricular zone‐olfactory bulb neurogenesis. Front Neurosci. 2014;8:142. 10.3389/fnins.2014.00142 24982610 PMC4058902

[54] Ernst A , Alkass K , Bernard S , et al. Neurogenesis in the striatum of the adult human brain. Cell. 2014;156:1072‐1083. 10.1016/j.cell.2014.01.044 24561062

[55] Nakatomi H , Kuriu T , Okabe S , et al. Regeneration of hippocampal pyramidal neurons after ischemic brain injury by recruitment of endogenous neural progenitors. Cell. 2002;110:429‐441. 10.1016/S0092-8674(02)00862-0 12202033

[56] Yamashita T , Ninomiya M , Acosta PH , et al. Subventricular zone‐derived neuroblasts migrate and differentiate into mature neurons in the post‐stroke adult striatum. J Neurosci. 2006;26:6627‐6636. 10.1523/JNEUROSCI.0149-06.2006 16775151 PMC6674034

[57] Boldrini M , Underwood MD , Hen R , et al. Antidepressants increase neural progenitor cells in the human hippocampus. Neuropsychopharmacology. 2009;34:2376‐2389. 10.1038/npp.2009.75 19606083 PMC2743790

[58] Campbell S , Marriott M , Nahmias C , MacQueen GM . Lower hippocampal volume in patients suffering from depression: a meta‐analysis. Am J Psychiatry. 2004;161:598‐607. 10.1176/appi.ajp.161.4.598 15056502

[59] Taylor WD , McQuoid DR , Payne ME , Zannas AS , MacFall JR , Steffens DC . Hippocampus atrophy and the longitudinal course of late‐life depression. Am J Geriatr Psychiatry. 2014;22:1504‐1512. 10.1016/j.jagp.2013.11.004 24378256 PMC4031313

[60] Vaccarino AL , Evans KR , Sills TL , Kalali AH . Symptoms of anxiety in depression: assessment of item performance of the Hamilton anxiety rating scale in patients with depression. Depress Anxiety. 2008;25:1006‐1013. 10.1002/da.20435 18800370

[61] Negoias S , Croy I , Gerber J , et al. Reduced olfactory bulb volume and olfactory sensitivity in patients with acute major depression. Neuroscience. 2010;169:415‐421. 10.1016/j.neuroscience.2010.05.012 20472036

[62] Croy I , Negoias S , Symmank A , Schellong J , Joraschky P , Hummel T . Reduced olfactory bulb volume in adults with a history of childhood maltreatment. Chem Senses. 2013;38:679‐684. 10.1093/chemse/bjt037 24051351

[63] Gaspersz R , Lamers F , Kent JM , et al. Longitudinal predictive validity of the DSM‐5 anxious distress specifier for clinical outcomes in a large cohort of patients with major depressive disorder. J Clin Psychiatry. 2017;78:207‐213. 10.4088/JCP.15m10221 27035515

[64] Siopi E , Denizet M , Gabellec MM , et al. Anxiety‐ and depression‐like states lead to pronounced olfactory deficits and impaired adult neurogenesis in mice. J Neurosci. 2016;36:518‐531. 10.1523/JNEUROSCI.2817-15.2016 26758842 PMC6602024

[65] Lau BWM , Yau SY , Lee TMC , Ching YP , Tang SW , So KF . Effect of corticosterone and paroxetine on masculine mating behavior: possible involvement of neurogenesis. J Sex Med. 2011;8:1390‐1403. 10.1111/j.1743-6109.2010.02081.x 20955318

[66] Martisova E , Aisa B , Tordera RM , Puerta E , Solas M , Ramirez MJ . Venlafaxine reverses decreased proliferation in the subventricular zone in a rat model of early life stress. Behav Brain Res. 2015;292:79‐82. 10.1016/j.bbr.2015.05.059 26051818

[67] Chen H , Pandey GN , Dwivedi Y . Hippocampal cell proliferation regulation by repeated stress and antidepressants. NeuroReport. 2006;17:863‐867. 10.1097/01.wnr.0000221827.03222.70 16738477

[68] Sousa N , Almeida OFX . Disconnection and reconnection: the morphological basis of (mal)adaptation to stress. Trends Neurosci. 2012;35:742‐751. 10.1016/j.tins.2012.08.006 23000140

[69] Bessa JM , Morais M , Marques F , et al. Stress‐induced anhedonia is associated with hypertrophy of medium spiny neurons of the nucleus accumbens. Transl Psychiatry. 2013;3:e266. 10.1038/tp.2013.39 23736119 PMC3693402

[70] Lopes S , Teplytska L , Vaz‐Silva J , et al. Tau deletion prevents stress‐induced dendritic atrophy in prefrontal cortex: role of synaptic mitochondria. Cereb Cortex. 2016;27(4):2580–2591. 10.1093/cercor/bhw057 27073221

[71] Morais M , Santos PAR , Mateus‐Pinheiro A , et al. The effects of chronic stress on hippocampal adult neurogenesis and dendritic plasticity are reversed by selective MAO‐A inhibition. J Psychopharmacol. 2014;28:1178‐1183. 10.1177/0269881114553646 25315831

[72] Morris M , Maeda S , Vossel K , Mucke L . The many faces of tau. Neuron. 2011;70:410‐426. 10.1016/j.neuron.2011.04.009 21555069 PMC3319390

[73] Fuster‐Matanzo A , de Barreda EG , Dawson HN , Vitek MP , Avila J , Hernández F . Function of tau protein in adult newborn neurons. FEBS Lett. 2009;583:3063‐3068. 10.1016/j.febslet.2009.08.017 19695252

[74] Kalil K , Dent EW . Branch management: mechanisms of axon branching in the developing vertebrate CNS. Nat Rev Neurosci. 2014;15:7‐18. 10.1038/nrn3650 24356070 PMC4063290

[75] Yuan A , Kumar A , Peterhoff C , Duff K , Nixon RA . Axonal transport rates in vivo are unaffected by tau deletion or overexpression in mice. J Neurosci. 2008;28:1682‐1687. 10.1523/JNEUROSCI.5242-07.2008 18272688 PMC2814454

[76] Criado‐Marrero M , Sabbagh JJ , Jones MR , Chaput D , Dickey CA , Blair LJ . Hippocampal neurogenesis is enhanced in adult tau deficient mice. Cells. 2020;9:210. 10.3390/cells9010210 PMC7016791 31947657

[77] Breuzard G , Pagano A , Bastonero S , et al. Tau regulates the microtubule‐dependent migration of glioblastoma cells via the Rho‐ROCK signaling pathway. J Cell Sci. 2019;132:jcs222851. 10.1242/jcs.222851 30659115

[78] Reiner O , Shmueli A , Sapir T . Neuronal migration and neurodegeneration: 2 Sides of the same coin. Cereb Cortex. 2009;19:i42‐i48. 10.1093/cercor/bhp039 19346270

[79] Hiesberger T , Trommsdorff M , Howell BW , et al. Direct binding of Reelin to VLDL receptor and ApoE receptor 2 induces tyrosine phosphorylation of Disabled‐1 and modulates tau phosphorylation. Neuron. 1999;24:481‐489. 10.1016/S0896-6273(00)80861-2 10571241

[80] Brich J , Shie FS , Howell BW , et al. Genetic modulation of tau phosphorylation in the mouse. J Neurosci. 2003;23:187‐192. 10.1523/jneurosci.23-01-00187.2003 12514215 PMC6742161

[81] Roberson ED , Scearce‐Levie K , Palop JJ , et al. Reducing endogenous tau ameliorates amyloid beta‐induced deficits in an Alzheimer's disease mouse model. Science. 2007;316:750‐754. 10.1126/science.1141736 17478722

[82] Bi M , Gladbach A , Van Eersel J , et al. Tau exacerbates excitotoxic brain damage in an animal model of stroke. Nat Commun. 2017;8(1):473. 10.1038/s41467-017-00618-0 28883427 PMC5589746

[83] Takahata K , Kimura Y , Sahara N , et al. PET‐detectable tau pathology correlates with long‐term neuropsychiatric outcomes in patients with traumatic brain injury. Brain. 2019;142:3265‐3279. 10.1093/brain/awz238 31504227

[84] Cho J‐H , Johnson GVW . Primed phosphorylation of tau at Thr231 by glycogen synthase kinase 3beta (GSK3beta) plays a critical role in regulating tau's ability to bind and stabilize microtubules. J Neurochem. 2004;88:349‐358. 14690523 10.1111/j.1471-4159.2004.02155.x

[85] Sotiropoulos I , Galas M‐C , Silva JM , et al. Atypical, non‐standard functions of the microtubule associated Tau protein. Acta Neuropathol Commun. 2017;5:91. 10.1186/s40478-017-0489-6 29187252 PMC5707803

[86] Lesuis SL , Hoeijmakers L , Korosi A , et al. Vulnerability and resilience to Alzheimer's disease: early life conditions modulate neuropathology and determine cognitive reserve. Alzheimers Res Ther. 2018;10(1):95. 10.1186/s13195-018-0422-7 30227888 PMC6145191

